# Diagnostic Potential of Ganglion Cell Layer to Inner Plexiform Layer Thickness Ratio in Distinguishing Branch Retinal Vein Occlusion from Primary Open-Angle Glaucoma

**DOI:** 10.21203/rs.3.rs-9944700/v1

**Published:** 2026-06-23

**Authors:** Rohith Erukulla, Moonis Ali, Neil Sheth, Jaden Wang, Deepak Edward, Darvin Yi, Jennifer Lim, Thasarat Vajaranant

**Affiliations:** University of Illinois at Chicago; University of Illinois at Chicago; University of Illinois at Chicago; University of Illinois at Chicago; University of Illinois at Chicago; University of Illinois at Chicago; University of Illinois at Chicago; University of Illinois at Chicago

**Keywords:** Branch Retinal Vein Occlusion, Primary Open-Angle Glaucoma, Optical Coherence Tomography, Ganglion Cell Complex, Inner Plexiform Layer

## Abstract

**Purpose:**

To investigate the diagnostic utility of the ganglion cell layer (GCL) to inner plexiform layer (IPL) thickness ratio, in differentiating branch retinal vein occlusion (BRVO) from primary open-angle glaucoma (POAG) exhibiting hemifield defects. Given the impact of POAG on the ganglion cell complex (GCC), we hypothesized that POAG wound show disproportionately greater thinning in the GCL and tested if the GCL:IPL ratio could differentiate between these two conditions.

**Methods:**

We conducted a retrospective case series of macular OCT images (Spectralis [Heidelberg Engineering, Heidelberg, Germany]) from patients with old BRVO/HRVO and POAG. Inclusion criteria were 1) BRVO/HRVO Diagnosis at least 6 months, without macular edema at the time of imaging, and 2) POAG with an arcuate or altitudinal hemifield defect on Humphrey Visual Field (Carl Zeiss Meditec, Inc., Dublin, CA). Exclusion criteria were the presence of poor-quality OCT scans, history of pan-retinal photocoagulation (PRP), corneal, retinal or neuroophthalmological conditions. Using the Heidelberg automated segmentation analysis of the macular cube, a 20-degree PMB grid was centered over the foveal pit and utilized to generate a 6×10 grid to provide a comprehensive assessment of the retinal layers in the macular region. The GCL:IPL ratio was calculated by dividing the GCL by the corresponding IPL thickness. Calculation of the inner retina:total retina ratio was done in an identical manner, and the average thicknesses and ratios were then compared using the Wilcoxon rank-sum test (MedCalc Statistical Software, Ostend Belgium).

**Results:**

Final analysis included 60 eyes of 60 patients (mean age 67 ± 13 years; 57% female; 42% African American, 28% Hispanic, 12% White, 18% as others) diagnosed with old BRVO/HRVO (n = 30) or POAG (n = 30). Patients with POAG had an average visual field mean deviation of −16.22dB ± 5.02. The GCL:IPL ratio was significantly lower in patients with POAG was 0.91 (95% CI [0.85, 0.94]) compared to RVO with 1.14 (95% CI [1.09, 1.17], (*P* < 0.0001). By adopting the GCL:IPL ratio of less than 1 as a diagnostic marker for POAG, the area under the curve (AUC) was 0.83, with a sensitivity of 90.0% and a specificity of 76.7%.

**Conclusions:**

There was disproportionately greater thinning in the GCL compared to the IPL in patients with POAG compared to those with RVO as evidenced by the observed differences in GCL:IPL ratios. Our findings characterized by high AUC and sensitivity suggest that the GCL:IPL ratio has potential to be a marker for distinguishing between old BRVO and POAG with hemifield defects.

## Introduction

Glaucoma is the leading cause of irreversible blindness, impacting over 70 million individuals worldwide [1]. Primary open-angle glaucoma (POAG) presents as a progressive optic neuropathy and is characterized by retinal ganglion cell death and subsequent visual field impairment. It is often associated with various comorbid conditions such as hypertension creating overlap with vascular pathologies such as retinal vein occlusions [2]. Glaucoma is also a known risk factor for retinal vein occlusions, which can present with similar clinical characteristics once past its acute phase [2, 3]. In brief, retinal vein occlusions can be further classified into branch retinal vein occlusions (BRVO), hemiretinal vein occlusions (HRVO), and central retinal vein occlusions (CRVO). In their acute phase retinal vein occlusions present with findings such as retinal hemorrhages, cotton wool spots, and macular edema along with changes in visual acuity [2, 4]. Once resolved, however, the presentation can be difficult to distinguish from glaucomatous retinal thinning and altitudinal defects.

Like POAG with hemifield defects, chronic BRVO and HRVO can present with hemifield defects and thinning of the inner retina on spectral-domain optical coherence tomography (SD-OCT. This similar clinical presentation combined with their co-occurrence can lead to diagnostic challenges and potential unnecessary treatment. The SD-OCT segmentation allows us to explore the distinct impact of pathophysiological mechanisms on various retinal layers, providing insights into their differential responses. For instance, previous research demonstrated that ganglion cell analysis (GCA) thickness maps generated from SD-OCT can provide a qualitative means of differentiating RVO from POAG based on patterns of thinning [6]. Specifically, use of the GCA thickness maps by independent examiners trained to identify RVO based on irregular topographic patterns of the thickness map produced a diagnostic specificity of 93.2% and diagnostic accuracy of 80.4%. However, the diagnostic utility of comparing specific retinal layers, particularly the GCL:IPL thickness ratio, has not been investigated. The difference in impact across the retinal layers secondary to the disease processes may be of utility in differentiating between the two. We hypothesized that unlike BRVO, which we expect thins both the Ganglion Cell Layer (GCL) and Inner Plexiform Layer (IPL), POAG would show disproportionately greater thinning in the GCL, and tested if the GCL:IPL ratio could differentiate between these two conditions. As such, we evaluated the diagnostic performance of the GCL:IPL thickness ratio, in differentiating BRVO from POAG with hemifield defects.

## Methods

This retrospective case-series study included patients with resolved BRVO and POAG with hemifield defects who visited the Illinois Eye and Ear Infirmary between 2014 and 2024. The study protocol adhered to the tenets of the Declaration of Helsinki and was approved by the Institutional Review Board of the University of Illinois.

### Subject Selection and Evaluation

Patients diagnosed with an old RVO (BRVO or HRVO) (n = 30) and POAG (n = 30) were reviewed in this study. For patients with old RVO, inclusion criteria consisted of at least 6 months past initial presentation, no macular edema at the time of imaging, and no concurrent glaucoma as identified by use of pressure lowering medications, surgery, or diagnosis. For patients with POAG, inclusion criteria consisted of a diagnosis of moderate or severe glaucoma with an arcuate or altitudinal hemifield defect on Humphrey Visual Field (Carl Zeiss Meditec, Inc., Dublin, CA). Exclusion criteria for all cases included poor-quality OCT scans (≤ 15dB signal strength), a history or pan-retinal photocoagulation (PRP), history of retinal detachment, concurrent uveitis, known corneal disease, other retinal pathology, or neuroophthalmological conditions.

### Spectral Domain Optical Coherence Tomography (OCT)

Macular OCT images were obtained using the Heidelberg Spectralis Spectral Domain OCT system in conjunction with the Heidelberg Eye Explorer software [Heidelberg Engineering, Heidelberg, Germany]. Automated segmentation of the retinal layers was completed through the Heidelberg Eye Explorer software. In instances where automated segmentation did not align with the actual retinal layers, manual adjustments were made by the operator to ensure accuracy of segmentation. A 20-degree PMB grid centered over the foveal pit and utilized to generate a 6×10 grid to provide a comprehensive assessment of the retinal layers in the macular region. This measurement was repeated for each of the retinal layers to create thickness maps for the GCL, IPL, Inner Retina, and Total Retina thickness. The Ganglion Cell-Inner Plexiform Layer (GCIPL) thickness was calculated by adding the GCL thickness to the IPL thickness in the corresponding locations. Thickness measurements were then compared in the affected and unaffected regions as well as between groups. The GCL:IPL ratio and the Inner Retina: Total Retina ratio were subsequently calculated by dividing the respective thicknesses at the corresponding grid locations.

### Statistical Analysis

Univariate analysis was performed using MedCalc Statistical Software (MedCalc Statistical Software, Ostend, Belgium). Data was assessed for normalization using the Shapiro-wilk test. Central tendency and variability of continuous variables were characterized by the median and 95% confidence intervals. Categorical variables were analyzed using frequency tables and examined using the Chi-squared test. Differences in retinal layer thicknesses and ratios between disease groups were obtained using the Mann-Whitney U test for independent samples. Intra-individual comparison of affected hemisphere retinal layer thickness against the unaffected hemisphere was performed with the Wilcoxon-signed-rank test for paired data. Receiver operating characteristic (ROC) curve analysis was conducted using ≤ 1 in the GCL:IPL ratio as a diagnostic marker for POAG.

## Results

### Demographics:

Final analysis included 60 eyes of 60 patients (mean age 67 ± 13 years; 43% male; 42% African American, 28% Hispanic, 12% White, 18% as others). Table 1 describes the demographics of the study groups. The RVO group consisted of 30% males while the POAG group had a 57% male population (*P* = 0.0388). Patients with POAG had an average visual field mean deviation of −16.22dB ± 5.02. There was no statistically significant difference in age or race between two groups (p &table; 0.05). Among the cohort, 75% had hypertension and 37% had type 2 diabetes with no statistical difference in prevalence between the POAG and RVO groups.

The location of the affected hemisphere showed a notable distribution difference between the two groups. In BRVO, 18 patients had a superior retinal defect and 12 had inferior, while in POAG there were 7 patients with superior retinal defects (with inferior hemifield loss) compared to 23 patients with inferior retinal defects (*P* = 0.0043).

### Retinal Layer Thickness:

The GCIPL thickness, inner retinal thickness, total retinal thickness were found to be thinner in the affected hemispheres compared to unaffected. Additionally, there was a statistically significant difference in these measurements between the RVO and the POAG groups. Comparisons intra-individual showed statistically significant thinning in the affected hemispheres in both groups as displayed in Table 2. In RVO, the median GCL:IPL ratio in the affected hemisphere was 1.14 (95%CI [1.09, 1.17]), compared to the unaffected hemisphere median of 1.18 (95% CI [1.14, 1.19]) (*P* = 0.0148). In POAG, the median GCL:IPL ratio in the affected hemisphere was 0.91 (95%CI [0.85, 0.94]) compared to the unaffected hemisphere median of 1.00 (95%CI [0.958, 1.049]) (*P* = < 0.0001). Similar differences were observed in the GCIPL thickness, inner retinal thickness, and retinal thickness as well as the inner retina: total retina ratio. Table 3 shows the comparison of retinal thicknesses in the affected hemisphere between the two groups. The retinal thickness (GCIPL, inner retina and total retina) was statistically significantly thinner in the POAG, compared to those with RVO. The marked difference was found in the GCIPL thickness with median RVO thickness being 65.2μm (95% CI [59.2, 68.2]) and the median in POAG being 44.1μm (95% CI [42.5, 44.7]). In addition, the median GCL:IPL ratio is statistically significantly higher in RVO (1.14 (95% CI [1.09, 1.17]), compared to POAG (0.91 (95% CI [0.85, 0.94]) (*P* = < 0.0001)). By adopting a GCL:IPL ratio less than 1 as a diagnostic marker for POAG, the area under the curve (AUC) was 0.833, with a sensitivity of 90.0% and a specificity of 76.7%.

## Discussion

Similarities in clinical presentation of historical RVOs and POAG present a diagnostic challenge. Appropriate identification is essential to prevent unnecessary treatment given the overlap often seen in these conditions. This investigation found three key findings. First, the GCIPL, Inner Retina, Total Retina were thinner in the affected area, compared to the unaffected area in both RVO and POAG. Second, the GCIPL, Inner Retina, Total Retina in the affected area were thinner in POAG with advanced hemifield defects, compared to RVO. Third, significant differences were observed in the GCL:IPL thickness ratios between the two disease groups in the affected area as well as in unaffected areas. Using an ROC curve, we found that using a GCL:IPL ratio of < 1 for POAG demonstrated a sensitivity of 90.0% and specificity of 76.6%. Demonstration of this uneven distribution in thinning, greater in the GCL, potentially serves as a promising marker to differentiate advanced POAG from RVO.

Studies have previously demonstrated retinal thinning, including the GCIPL thickness, in both patients with RVO and in POAG [8, 10, 12]. We observed these changes in both RVO and POAG patients in the GCIPL, the inner retina, and the total retina. The greater degree of thinning observed in the affected regions also supports the resolution of macular edema in the patients with RVO. As the GCIPL layer thins we hypothesized that the thinning is disproportionately greater in POAG compared to RVO. Moghimi and colleagues in 2019 found that there was no preferential thinning of the IPL layer in glaucoma when compared with the GCL [11], which we contrast with the expected change in the IPL in RVO secondary to the ischemic changes and macular edema. Given that both pathologies displayed thinning across the analyzed layers, we compared the amount of thinning in the affected area between groups and found greater thinning across the layers in advanced POAG. Our finding contradicts what was found in a previous study that compared the average GCIPL thickness between RVO and POAG, however this may be explained by the fact that that the previous study matched RVO patients with glaucoma patients based on average RNFL thickness (within ± 5 μm) [6]. Our study focused on glaucomatous patients with the presence of advanced hemifield defects as evidenced by the average mean deviation of −16.22dB ± 5.02. Both studies assessed RVO patients with resolved macular edema and at least 6 months past the acute phase.

Mahmoudinezhad and colleagues studied the utility of ganglion cell layer (GCL) and the ganglion cell complex (GCC) for the detection of early glaucoma. They identified similar performance in detection of early glaucoma [10]. We utilized the GCL in conjunction with the IPL to generate a ratio to differentiate RVO from POAG which, given the best of our knowledge, no study has investigated. The GCL:IPL ratio in patients with BRVO/HRVO was on average ≥ 1, while the POAG GCL:IPL ratio was ≤ 1 within the affected hemisphere. This finding supports our initial hypothesis that there is disproportionately greater thinning of the GCL in POAG patients compared to those with RVO. Due to this, we adopted a GCL:IPL ratio of less than 1 as a diagnostic marker for POAG and found to be a promising marker for identification of POAG between these disease processes. Distinguishing between an old BRVO/HRVO and POAG is imperative for medical decision-making and initiation of appropriate medical or surgical therapies.

The demographic-matched groups reduced concerns of confounding variables such as IPL thinning associated with race [11] and strengthened the overall study. However, there are some limitations to this study including the retrospective nature of its design. First, the RVO data had significantly more variation which could be in part due to the variance in presentation. A potential source of this is the varied presentations of RVO which may benefit from further stratification through characterization such perfused vs non-perfused. Second, thinning of the retinal layers, disorganization of retinal inner layers (DRIL) presence in RVOs [5], and scan quality can lead to inaccuracies in automated segmentation of the retinal layers. To minimize this, we manually adjusted segmentation as needed. Third, the co-occurrence of the two disease pathologies lends itself to the risk that the eyes studied have undiagnosed overlapping pathologies. Studies with larger sample sizes and a healthy eye group may provide greater granularity in differentiating the groups.

In conclusion, there was disproportionately greater thinning in the GCL compared to the IPL in patients with POAG compared to those with RVO as evidenced by the observed differences in GCL:IPL ratios. This was observed in comparison of the affected areas between the two groups as well as comparing the change observed between affected and unaffected hemisphere. Our findings characterized by high AUC and sensitivity suggest that the GCL:IPL ratio has potential to be a marker for distinguishing between old BRVO and advanced POAG. Future studies are needed to validate and refine the clinical utility of the GCL:IPL ratio.

## Figures and Tables

**Table 1.** Demographics and clinical characteristics

	Retinal Vein Occlusion (n=30)	Primary Open-Angle Glaucoma (n=30)	p-Value
Age, y (SD)	68.57±11.04	64.63±13.83	0.2276
Sex, male %	30%	57%	0.0388
Affected Hemisphere			0.0043
Superior	18	7	
Inferior	12	23	
Hypertension %	80%	70%	0.3751
Type 2 Diabetes %	33%	40%	0.5952

Chi-Squared test used for characteristic variables; Independent T-test used for continuous variables.

**Table 2.** Comparative changes between affected and unaffected hemisphere retinal layer thickness in retinal vein occlusion and primary open-angle glaucoma.

	Affected	Unaffected	p-Value
**RVO (n=30)**			
GCIPL Thickness, μm (95%CI)	65.2 [59.1, 68.2]	71.2 [67.0, 73.3]	0.0008
Average Inner Retinal Thickness, μm, (95%CI)	215.8 [209.2, 221.7]	225.1 [218.9, 230.1]	0.0026
Average Retinal Thickness, μm (95%CI)	294.6 [287.8, 303.3]	303.0 [299.8, 307.2]	0.0037
GCL:IPL (95%CI)	1.14 [1.09, 1.17]	1.18 [1.14, 1.19]	0.0148
Inner Retina: Total Retina (95%CI)	0.73 [0.72, 0.74]]	0.737 [0.73, 0.74]	0.0020
**POAG (n=30)**			
GCIPL Thickness, μm (95%CI)	44.1 [42.5,44.7]	48.17 (45.31,51.37)	0.0002
Average Inner Retinal Thickness, μm, (95%CI)	193.1 [186.5, 199.0]	205.8 [191.0, 214.8]	0.0005
Average Retinal Thickness, μm (95%CI)	271.1 [265.8, 278.1]	285.2 [267.1, 294.1]	0.0010
GCL:IPL (95%CI)	0.91 [0.85, 0.94)	0.997 [0.96, 1.05]	<0.0001
Inner Retina: Total Retina (95%CI)	0.71 [0.70, 0.71]	0.720 [0.71, 0.73]	0.0006

Wilcoxon Test (paired samples), GCIPL = Ganglion Cell-Inner Plexiform Layer, GCL = Ganglion Cell Layer, IPL = Inner Plexiform Layer

**Table 3.** Comparative changes in retinal layer thicknesses in areas of affected retina.

	Retinal Vein Occlusion (n=30)	Primary Open-Angle Glaucoma (n=30)	p-Value
GCIPL Thickness, μm (95%CI)	65.2 [59.2, 68.2]	44.1 [42.5, 44.7]	<0.0001
Average Inner Retinal Thickness, μm (95%CI)	215.8 [209.2, 221.7]	193.1 [186.5, 199.0]	0.0007
Average Total Retinal Thickness, μm (95%CI)	294.6 [287.8, 303.3]	271.1 [265.8, 278.1]	0.0007
GCL:IPL (95%CI)	1.14 [1.09, 1.17]	0.91 [0.85, 0.94]	<0.0001
Inner Retina: Total Retina (95%CI)	0.73 [0.721, 0.737]	0.71 [0.70, 0.71]	0.0007

Mann-Whitney-U Test (independent samples), GCIPL = Ganglion Cell-Inner Plexiform Layer, GCL= Ganglion Cell Layer, IPL = Inner Plexiform Layer

## Data Availability

The data that support the findings of this study will be shared upon reasonable request.
